# Impact of sea level rise on the Mediterranean *Lithophyllum byssoides* rims

**DOI:** 10.1038/s41598-023-37110-3

**Published:** 2023-06-29

**Authors:** Aurélie Blanfuné, Charles-François Boudouresque, Marc Verlaque, Antoine Minne, Fanny Noisette, Thierry Thibaut

**Affiliations:** 1 Aix Marseille University, Université de Toulon, CNRS, IRD, MIO (Mediterranean Institute of Oceanography), UM 110, 13288 Marseille, France; 2 Oceans Institute and School of Biological Sciences, University of Western Australia, Crawley, WA Australia; 3 Institut Des Sciences de La Mer, Université du Québec À Rimouski, Rimouski, QC G5L 3A1 Canada

**Keywords:** Conservation biology, Ecology

## Abstract

The calcified red macroalga *Lithophyllum byssoides*, a very common midlittoral species in the western Mediterranean Sea, is a significant ecosystem engineer capable, under exposed and dim light conditions, of building wide and solid endemic bioconstructions near the mean sea level: the *L. byssoides* rims or '*trottoirs à L. byssoides'*. Although the growth of the species is relatively rapid for a calcified alga, the construction of a large rim requires several centuries of near stable or slowly rising sea level. As the time scale of their formation is measured in centuries, *L. byssoides* bioconstructions constitute valuable and sensitive sea level markers. The health status of *L. byssoides* rims has been studied at two sites located far apart from each other (Marseille and Corsica), both in areas heavily impacted by humans and in areas with little impact (MPAs and unprotected areas). A health index is proposed: *Lithophylum byssoides* Rims Health Index. The main and inevitable threat is the rise in the sea level. This ecosystem would be the first case worldwide of marine ecosystem collapse resulting, indirectly, from man-induced global change.

## Introduction

After the coral-reef scleractinians, calcified red algae are the most significant ecosystem engineers building solid bioconstructions. Despite their ecological value, a recent review assessing the research efforts focused on Mediterranean bioconstructions identified *Lithophyllum* rims as being the least studied and highlights the overall neglected status of these ecosystems^1^. Moreover, *Lithophyllum byssoides* (Lamarck) Foslie builds bioconstructions that are sensitive to sea level and can therefore be important indicator species in the face of sea level rise (e.g.^2–8^. By coalescence of individuals, the species can form more or less extensive calcareous bioconstructions, depending on the environmental conditions: (1) a few centimetres thick coating (< 5 cm thick) on gently sloping rocks, (2) swellings on vertical rocks (thicker than coatings, but without an overhang), and, (3) when conditions are particularly favourable (shady coves exposed to surf action), rims more than 20 cm wide and presenting at least the beginning of an overhang. Locally, *L. byssoides* rims can reach up to 2 m wide^9–14^. This intertidal ecosystem produces biogenic concretions housing a high algal and invertebrate biodiversity. These calcareous bioconstructions protect the underlying rock surfaces from the action of bio-eroders such as endolithic Cyanobacteria and Chlorophyceae, clinoid sponges and the boring mussel *Lithophaga lithophaga* (Linnaeus, 1758)^15^, but also allow their installation in siliceous regions^14^. The inner structure of *L. byssoides* rims revealed consolidated layers of dead thalli and the core has a multi-layered structure with numerous discontinuities^6^. These algal rims shelter numerous populations of bivalves e.g., *Mytilaster lineatus* (Gmelin, 1791) and *Lasaea* spp.^6^. This ecosystem occupies an intermediate position between the marine and the terrestrial environments. Larval stages of *Clunio boudouresquei* Moubayed-Breil, 2019 (Diptera: Chironomidae) are exclusively confined to the *Lithophyllum* rims^16^.

Although the growth of the species is relatively rapid for a calcified alga^17^, the construction of a large rim requires several centuries of near stable or slowly rising sea level^3,4,14,18^. The time scale of their formation is therefore measured in centuries. For example, the age of the *L. byssoides* rim at Cala Litizia, in the Scandola Nature Reserve (hereafter SNR, western Corsica), has been estimated to be between 900 and 1000 years^3^. *Lithophyllum byssoides* is sensitive to coastal disturbances (sea surface pollution, trampling, etc.). The lifespan of *L. byssoides* rims makes them good indicators of long periods of stable or slowly rising sea level as they cannot tolerate being permanently submerged. *Lithophyllum byssoides* rims are stable over a short timescale, but their health can change at decadal scale. For example, in the SNR, since the first exhaustive mapping of *L. byssoides* (coatings, swellings and rims) carried out between 1981 and 1986^13^, several signs of degradation have been observed: decrease in surface area of living *L. byssoides*, presence of traces of human mechanical impact, alveolar bioerosion and development of an epibiontic algal cover, in particular articulated coralline algae^20,21^. The ongoing deterioration of this unique ecosystem may be caused by a wide range of suspected factors. Trampling is probably a rare cause of deterioration of *L. byssoides* rims because of their situation in areas inaccessible for disembarkation and their width, which is too narrow for walking. Some of these signs of degradation have been attributed to the global sea level rise: the upper shoulder of the *L. byssoides* rim cannot grow upward as fast as the sea level rise, so that it ends up being in the infralittoral zone (subtidal zone) while it is strictly linked to the mediolittoral zone^3,4,22,23^ (see Pérès and Picard^24^, for the delineation of midlittoral and infralittoral zones in the Mediterranean Sea). Their precisely limited vertical range in sheltered locations (± 10 cm) comes from the restricted environmental niche of *L. byssoides* that exclusively inhabits a narrow part of the intertidal zone. The lower limit of these bioconstructions is defined as biological mean sea-level^2,4,19–21,25,26^.

Because of its ecological importance, *L. byssoides* and its bioconstructions appear, under an old algal synonym *L. lichenoides*, in the list of species of Appendix I of the Bern Convention^27^, and in that of Appendix II of the Barcelona Convention (protocol relating to specially protected areas and biological diversity in the Mediterranean Sea), as association of *Lithophyllum lichenoides* Philippi or *Lithophyllum lichenoidis* Giaccone 1993^28^. In the European EUNIS classification, it is recognised in the II.4.2.1 Association with *Lithophyllum byssoides*^29^. *L. byssoides* rims are also used as indicators in European Directives (Water Framework Directive, WFD and Marine Strategy Framework Directive, MSFD). Along the French Mediterranean coast, *L. byssoides* is widely distributed on the rocky coasts of Corsica, French Riviera and Provence, but is uncommon in Occitania, where most of the shoreline is sandy. Where *L. byssoides* is present, rims are confined to very localized suitable sites. The most recent exhaustive mapping was carried out in the framework of the assessment of the macroalgal descriptor of the European Union WFD, where *L. byssoides* rims of the French mainland coast were mapped: their accumulated length was 36 km (at a scale of 1/2500), which represents 2.4% of the shoreline^30–37^.

The lower limit of *L. byssoides* can be considered as one of the most precise relative sea-level markers in the Mediterranean, due to its inability to withstand permanent immersion^4,6,26^. Consequently, on the basis of an accurate and reproducible protocol, we have proposed a health index, the *Lithophylum byssoides* Rims Health index (LBRHI), to assess the vitality of the *L. byssoides* rims in the context of the rise in sea level and to serve as a record of sea level in the perspective of global change. We have assessed and analysed the health status of *L. byssoides* rims within and off two northwestern Mediterranean MPAs (Marine Protected Areas), the first in Corsica, the SNR in the Regional Natural Park of Corsica, established in 1975^38^, and the surrounding area, and the second in Provence, near Marseille (southern France), the Calanques National Park (CNP), established in 2012. We also monitored the degradation of the Cala Litizia rim by comparing historical data from 1995 to a recent survey from 2014 to track the evolution of its health status and implemented the *Lithophylum byssoides* Rims Health Index (LBRHI) in all surveys referred to in this study.

## Material and methods

The protocol for quantifying the state of *L. byssoides* rims was adapted from the method proposed and tested in 1995 by Verlaque^21^ and published in 2010^26^. The Verlaque protocol was based upon the relative frequency of the different taxa dwelling on the upper surface of a rim, estimated in situ by the ‘point intercept’ method (20 × 20 cm or 40 × 40 cm quadrats including 100 intercepts were used according to the width of *L. byssoides* rims).

The new protocol was based upon a photographic survey carried out over the entire bioconstruction. For each rim, a photographic survey of the upper surface of a significant portion (several linear meters, up to 90%) was performed. All the images were analysed in the laboratory, using image processing software (Image J®), and the percent cover of different items (taxons and holes—see below) was assessed. Only the usable parts of the photos (non-blurred, non-submerged), which differ from one photo to another and from one site to another (Table 1), were analysed. In order not to bias the comparison between 1995^21^ and 2014–2015 (this study), the method of analysis of the photos being slightly different, the Verlaque photos were re-analyzed using our method.

**Table 1:** Characteristics of the studied sites for the assessment of the status of *Lithophyllum byssoides* rims. M1 through M8: Marseille. C1 through C11: Corsica.

	Acronym	Site	Location	Hydrodynamism	Height (above mean sea level)	Coordinate X (decimal degrees)	Coordinate Y (decimal degrees)
Marseille (Provence)	M1	Calanque de l'Eissadon	Core of the MPA—R	High	Close	5.487887653	43.20408464
	M2	Calanque de l'Oule	Core of the MPA—R	High	Very high	5.491602675	43.20332067
	M3	Castel Vieil 1	Core of the MPA—R	High	Very high	5.496045000	43.19989500
	M4	Castel Vieil 2	Core of the MPA—R	High	Very high	5.494439739	43.20148357
	M5	Impérial du milieu	Core of the MPA—SP	High	High	5.393847690	43.17157339
	M6	Contrebandier 1	Core of the MPA—SP	Moderate	Close	5.385535488	43.17372185
	M7	Contrebandier 2	Core of the MPA—SP	Moderate	Close	5.385445540	43.17360889
	M8	Contrebandier 3	Core of the MPA—SP	moderate	Close	5.385921297	43.17402217
Scandola and surrounding area (Corsica)	C1	Palazzu Cave	Reserve	High	High	8.550510597	42.37805742
	C2	Cala Litizia	Reserve	Moderate	Close	8.546938671	42.37649284
	C2bis	Cala Litizia entrance	Reserve	High	Very high	8.546938671	42.37649284
	C3	Punta Palazzu	Reserve	High	High	8.545046310	42.37410877
	C4	Gattaghja South	Reserve	High	Close	8.557736580	42.33935357
	C5	Gattaghja North	Reserve	Moderate	Close	8.558045590	42.34282965
	C6	Palazzu Islet	Reserve	Moderate	Very high	8.546459913	42.38022104
	C7	Punta Rossa	Off Reserve	High	Close	8.606036198	42.41581741
	C8	Punta di U Stollu	Off Reserve	Moderate	Close	8.622743653	42.42148240
	C9	Gulf of Galeria 1	Off Reserve	High	High	8.627108326	42.42074306
	C10	Gulf of Galeria 2	Off Reserve	High	High	8.627584962	42.42027908
	C11	Gulf of Galeria 3	Off Reserve	Moderate	Close	8.630715151	42.42061269

The season for performing the photo assessment is crucial in terms of its quality as the appearance of *L. byssoides* varies with exposure to waves, temperature, and light. The method requires calm sea conditions. In winter, weather conditions are often too poor for good photographic coverage. In spring, seasonally developing ephemeral soft macroalgae can obscure the surface of the rim. Finally, in summer, high temperature, prolonged emersion and sunlight whiten *L. byssoides*, making it difficult to distinguish between living and dead parts. The best-suited field period is in the fall, when the living parts of *L. byssoides* are clearly visible and the soft macroalgae are poorly developed^26^.

**Figure 1:**
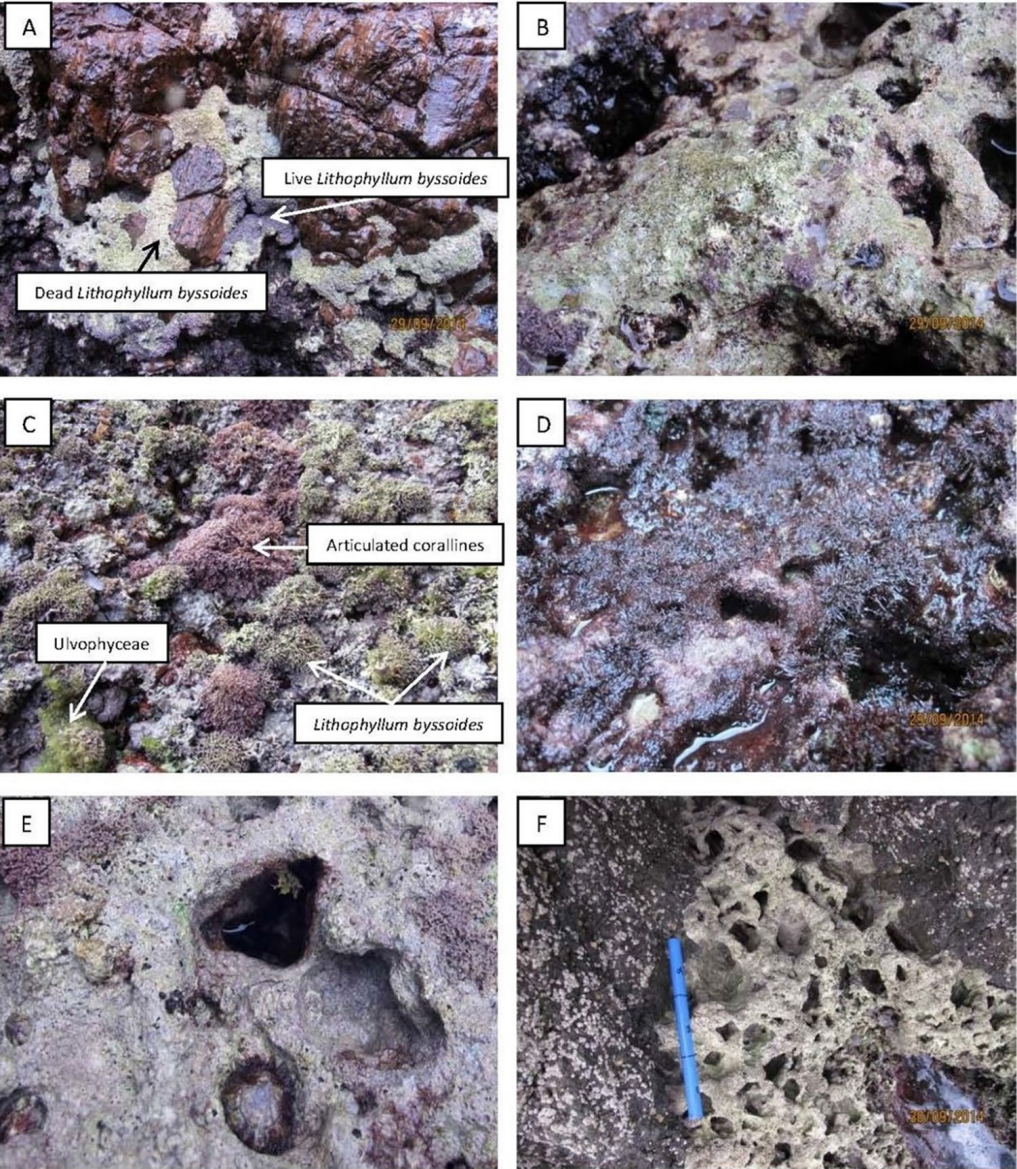
(**A**) Live (pink) and dead (white) *L. byssoides*. (**B**) Dead and eroded *L. byssoides*. (**C**) Live individuals of *L. byssoides,* with well-developed cristae, articulated corallines and Ulvophyceae. (**D**) Soft Rhodophyta. (**E**) Live crustose corallines and a hole. (**F**) Heavily bio-eroded *L. byssoides* rim, with many holes. Photos © Aurélie Blanfuné and Thierry Thibaut.

When analysing the photos, the seven following items were considered as descriptors (Fig. 1): (i) living parts of *L. byssoides*, i.e. in the form of bulges with visible and coloured ridges (live *L. byssoides*—LLB); (ii) dead parts of *L. byssoides* without epibionts, i.e. ridges discoloured (white), eroded or damaged (dead *L. byssoides*—DLB); (iii) articulated corallines (red algae, Florideophyceae) (Articulated corallines—COR); (iv) ephemeral green algae (Ulvophyceae) (Ulvophyceae—U); (v) soft red algae (Rhodophyta—RH); (vi) live crustose calcified red algae (Corallinales and Hapalidiales, Florideophyceae), i.e. smooth and red coloured surfaces without outgrowths (Crustose corallines—CC); these algae form a thin cover, which corresponds to the recruitment of the year of *L. byssoides*, in addition to the other live calcified species contributing to the cementation of the interstices between *L. byssoides* thalli, and therefore to the edification of the rim, e.g. *Neogoniolithon brassica-florida* (Harvey) Setchell & L.R. Mason; **(**vii**)** the holes (holes—H). Since the photographic coverage has been entirely analysed, the percentages of each category are representative of the health status of the bioconstruction.

Two of the seven descriptors of the *L. byssoides* rim reflect a good health status: the surface area covered by live *L. byssoides* (LLB) and live crustose corallines (CC). The other descriptors are indicators of its degradation: (1) dead *L. byssoides* (DLB); (2) articulated corallines (COR), infralittoral genera (*Corallina* and *Ellisolandia*) whose presence on the *L. byssoides* rim reflects the global sea level rise^39^; (3) ephemeral green algae (U), which reflect the death of the *L. byssoides* and the eutrophication of the water body; midlittoral green algae, whose presence is normal on the rim, e.g., *Bryopsis muscosa* J.V. Lamouroux, are poorly represented in autumn^40^; (4) soft Rhodophyta (RH), for the most infralittoral algae that also reflect the sea level rise; (5) holes (H) due to the bioerosion of the calcified bioconstruction of *L. byssoides* and other crustose corallines by clionid sponges, boring mollusks, e.g*. Lithophaga lithophaga* (Linnaeus, 1758), and endolithic cyanobacteria and Ulvophyceae^19,26^. Once a cup-shape cavity is initiated, water stagnation and humidity accelerate its enlargement; when the cavity pierces the rim, it becomes a blow hole, which is subsequently enlarged by the surf.

We propose a health index for assessing the status of *L. byssoides* rims, the *L. byssoides* Rims Health Index (LBRHI), based upon the above-mentioned descriptors, expressed as percent cover of the upper surface area of the rim:

$${\text{LBRHI}} = \left( {{\text{LLB}} + {\text{CC}}} \right)/\left( {{\text{LLB}} + {\text{CC}} + {\text{DLB}} + {\text{COR}} + {\text{U}} + {\text{RH}} + {\text{H}}} \right)$$

**Figure 2:**
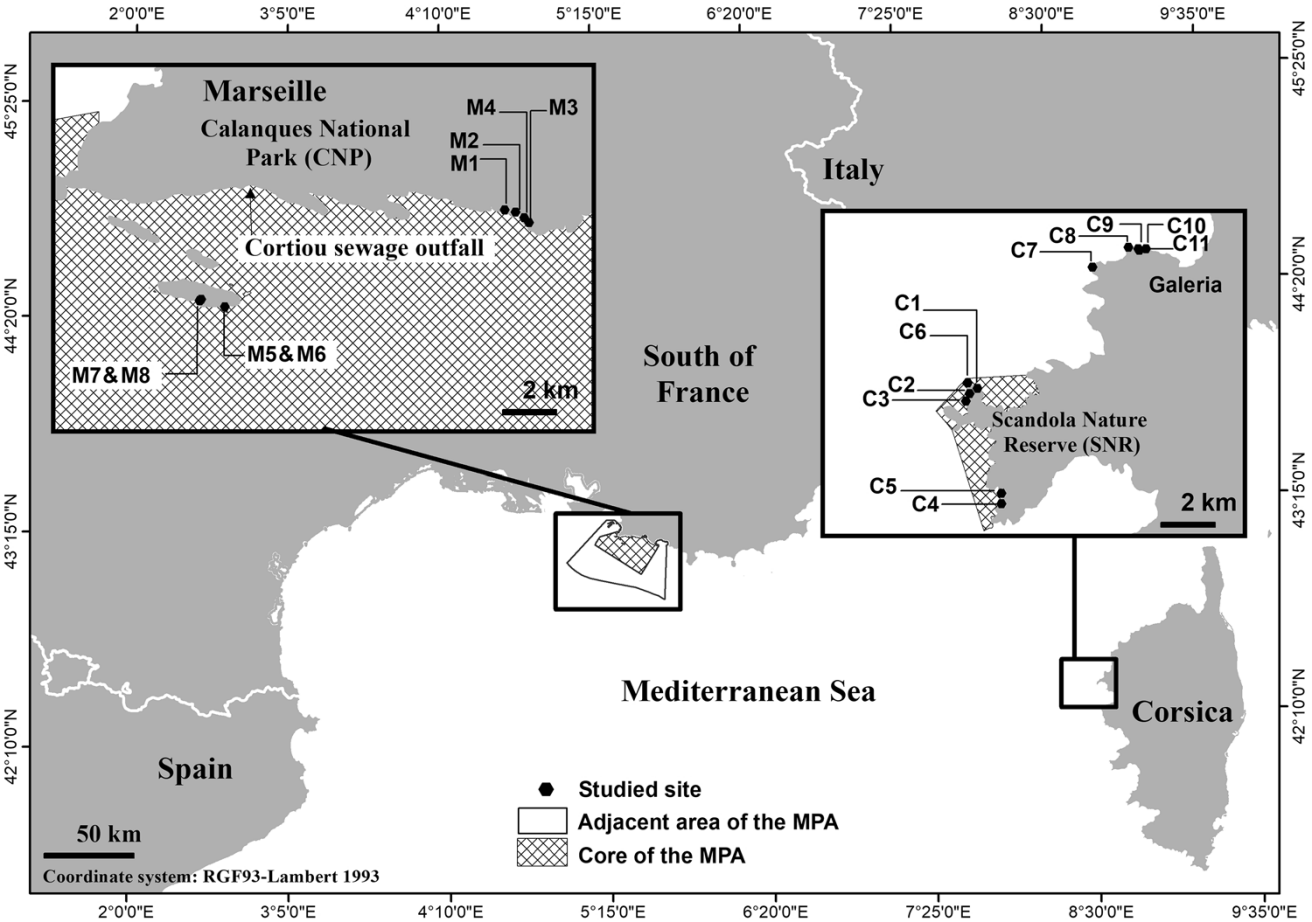
Location of studied sites for the assessment of the status of *Lithophyllum byssoides* rims during 2014 in Corsica and during 2015 in Marseille.

The LBRHI index corresponds to the sum of the percentages of the LLB + CC categories, (categories containing live *L. byssoides*) divided by the sum of the percentages of all the categories. Since the portion of the rim is analysed in its entirety, the sum percentages of each category correspond to the entire upper surface. Therefore, there is only one index per rim and the index (LBRHI) can take values between 0 and 1. The worst health status is indicated by 0, while the best is indicated by 1. Our study sites were selected according to the recent exhaustive mappings of *L. byssoides* rims^32–34,36^ (Table 1). They were chosen according to (1) the width and the presence of an overhang, corresponding to the definition we give of a *L. byssoides* rim; (2) their suitability and accessibility for fieldwork, with the possibility for researchers to make observations under acceptable conditions of safety; (3) the diversity of conditions of exposure to waves and light; (4) the height above mean sea level; (5) the diversity of morphology; and (6) the location in or off the core area of a MPA.The field survey was carried out from September 29 to October 1, 2014 in the SNR and surrounding area (Corsica) (6 fully protected and 5 unprotected sites) and in December 2015 in the CNP (Marseille) (4 strictly protected (SP—no fishery) and 4 regulated sites (R—regulated artisanal fishing only)) (Table 1**, **Fig. 2).

LBRHI differences between study areas (Marseille vs Corsica) and protection status (protected vs non-protected) were tested with Student’s t test, after verifying data normality.

PERMutational univariate Analyses of Variance (PERMANOVA), MultiDimensional Scaling (MDS) multivariate analyses and Distance-based redundancy analysis (*dbRDA)* were performed on the descriptors (percentage of coverage of the following categories: live *Lithophyllum byssoides*, *L. byssoides *dead, articulated corallines, Ulvophyceae, Rhodophyta, live crustose corallines and holes) and site characteristics (MPA or outside MPA; the height of the *L. byssoides* rim in relation to the mean sea level: close to sea level (Low), high, very high) using PRIMER software. The data has undergone a square root transformation. The similarity matrix was performed using S17 Bray Curtis to represent the distribution of samples in a space defined by different variables^41,42^. The contribution of the variables used has been represented, in parallel, on a correlation circle.

## Results

### Assessment of the status of *Litophyllum byssoides* rims

Overall, the health status of *L. byssoides* rims in the two study areas, Marseille and Corsica, is poor (mean LBRHI = 0.25 ± 0.04, SD = 0.16), with a LBRHI index ranging between 0.11 and 0.69 in Marseille (mean = 0.34 ± 0.07, SD = 0.20), and only between 0.03 and 0.32 in Corsica (mean = 0.18 ± 0.03, SD = 0.10) (Table 2). The status is worse in the SNR (Scandola, Corsica) than in CNP (Marseille) (student’s t test = 5.99***; *df* = 17), with the highest LBRHI values mostly found in offshore/island sites in Marseille (M6, M7 and M8, Table 2).

**Table 2:** Assessment of the status of the studied *Litophyllum byssoides* rims (percentage of coverage established by photographic analysis).

	Acro-nym	Site	Surface analysed (cm^2^)	Live *Litophyllum byssoide*s (LLB)	Dead *Litophyllum byssoides* (DLB)	Articulated corallines (COR)	Ulvo-phyceae (U)	Rhodo-phyta (RH)	Live crustose corallines (CC)	Holes (H)	LBRHI index
Marseille (Provence)	M1	Calanque d'Eissadon	16,080	10.8	26.1	1.3	1.1	31.3	22.7	6.7	0.34
	M2	Calanque d'Oule	8185	0.3	66.4	1.1	0.2	9.4	17.1	5.4	0.17
	M3	Castel Vieil 1	6053	6.0	42.6	17.0	5.9	22.2	4.6	1.6	0.11
	M4	Castel Vieil 2	11,272	6.8	47.3	2.9	2.8	25.6	9.2	5.4	0.16
	M5	Impérial du milieu	9547	22.2	47.9	4.7	1.5	8.6	8.0	7.2	0.30
	M6	Contrebandier 1	11,162	47.6	12.2	0.9	0.7	15.3	21.2	2.2	0.69
	M7	Contrebandier 2	10,444	28.8	17.2	10.9	2.3	15.6	13.2	12.1	0.42
	M8	Contrebandier 3	7493	28.7	17.4	10.6	1.0	15.0	21.2	6.1	0.50
Scandola and surrounding area (Corsica)	C1	Palazzu Cave	8058	1.3	60.3	0.0	0.1	6.8	8.5	23.0	0.10
	C2	Cala Litizia	8716	22.4	33.5	8.1	0.0	15.0	9.4	11.7	0.32
	C3	Punta Palazzu	8076	18.6	38.5	0.0	19.3	1.0	8.0	14.6	0.27
	C4	Gattaghja South	6175	18.2	52.2	1.7	0.3	14.9	6.4	6.3	0.25
	C5	Gattaghja North	8800	8.8	63.4	0.0	0.0	0.0	7.2	20.6	0.16
	C6	Palazzu Islet	9278	26.7	52.8	0.0	0.3	0.7	5.3	14.2	0.32
	C7	Punta Rossa	9726	1.0	89.6	0.0	1.5	0.1	2.1	5.7	0.03
	C8	Punta di U Stollu	8963	5.4	64.1	1.2	0.6	14.4	7.0	7.2	0.12
	C9	Gulf of Galeria 1	9378	7.5	74.5	0.0	0.8	1.2	7.9	8.1	0.15
	C10	Gulf of Galeria 2	9672	6.3	77.6	0.0	0.4	0.5	5.1	10.1	0.11
	C11	Gulf of Galeria 3	9313	11.0	76.8	0.0	0.4	1.4	4.2	6.2	0.15

The percentage of live *L. byssoides* is in some cases very low: 0.3%, 1.3%, 1.0% and 5.4%, for sites M2, CA, C7 and C6, respectively. The percentage of dead *L. byssoides* is often very high: 66.4%, 89.6%, 74.5%, 77.6% and 76.8% at M2, C7, C9, C10 and C11, respectively. The holes exceed 20% of the surface area of the rim at sites C1 and C5. Infralittoral seaweeds, evidence of the rise in sea level, may cover up to 45% of the upper surface at M3.

### The Cala Litizia rim (Scandola): a forty-year follow up (1975 to 2014)

**Figure 3:**
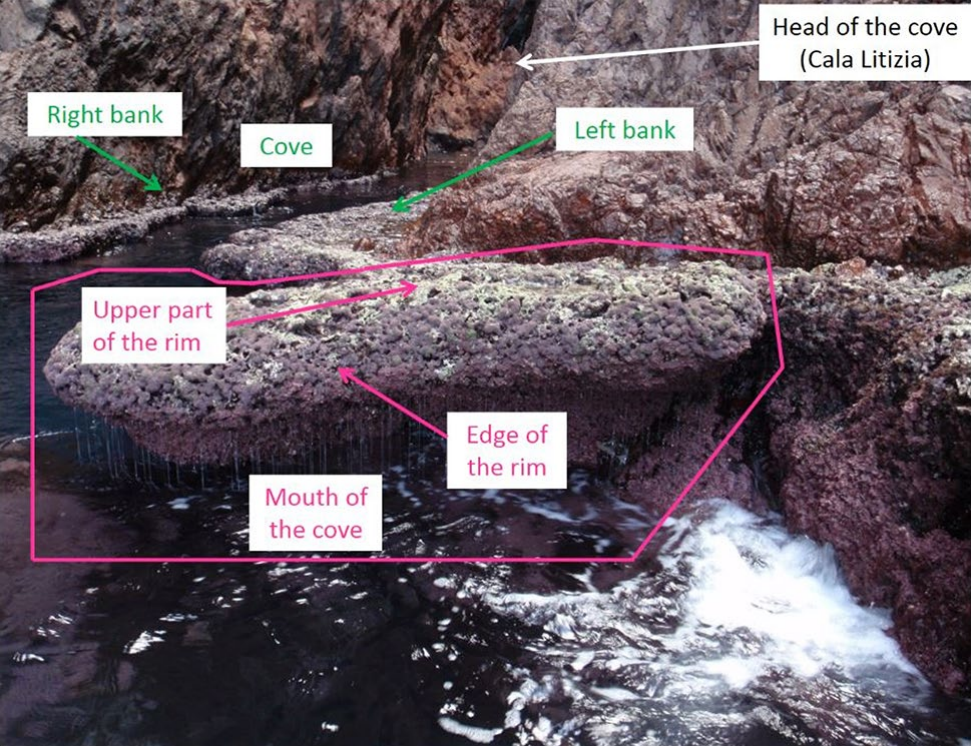
The *Lithophyllum byssoides* rim of Cala Litizia (SNR, Corsica), seen from the sea. Photo @Thierry Thibaut.

Between 1992 and 1995, the state of the rim became deteriorated: the surface area of live *L. byssoides* decreased (dark pink or dark purple in Verlaque’s photos), the number and size of the holes increased, and the near absence of signs of recovery was noted compared with photos taken three years earlier^21^. In 2014 (this study; Fig. 3), the percentage of live *L. byssoides* fell to 22.4% (Table 2). At the same time, dead *L. byssoides*, infralittoral macroalgae (COR, RH), Ulvophyceae (U), and holes almost doubled their percent cover. This regressive trend concerned all the parts of the rim, with the exception of the seaward edge of the rim, at the mouth of the cove (Table 3). In contrast with 1995, when no gradient of degradation was apparent, the 2014 health status of the *L. byssoides* rim declined from the mouth to the head of the cove, until it was completely submerged.

**Table 3:** Changes in the surface composition of the Cala Litizia rim between 1995 and 2014 (percentage of cover established by photographic analysis). See Fig. 4 for the localization of the study areas.

	Year	Live *L. byssoides* (LLB)	Dead *L. byssoides* (DLB)	Articulated corallines and Rhodophyta (COR + RH)	Ulvo-phyceae (U)	Live crustose corallines (CC)	Holes (H)	LBRHI index
Edge (mouth of the cove)	1995	65.5	18.3	0.9	4.1	4.5	6.7	0.70
	2014	56.5	12.8	0.2	1.9	20.3	8.3	0.77
Upper part (mouth of the cove)	1995	65.1	18.5	0	0.2	0	16.2	0.65
	2014	32.1	28.9	12.8	0.2	6.7	19.3	0.39
Right bank of the cove	1995	54.4	38	0	0	0	7.7	0.54
	2014	28.9	37.5	3.9	0	9.2	20.4	0.38
Left bank of the cove	1995	47.0	28.7	0	0	0	24.3	0.47
	2014	16.0	34.1	37.8	0	0	12.1	0.16

*Data for 2014 relate only to the most offshore part of the bank, which is not yet submerged.

**Figure 4:**
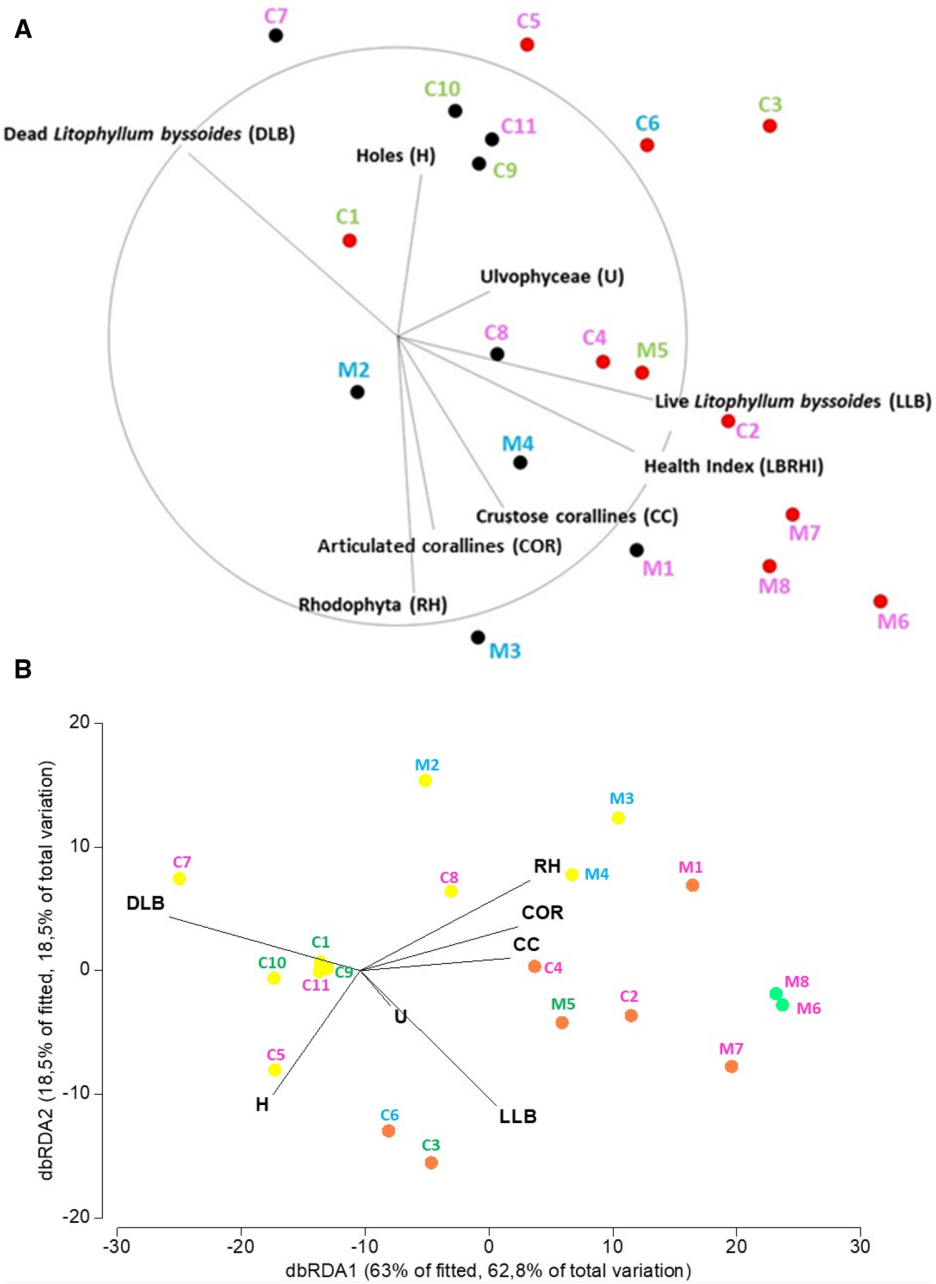
**(A)** MDS (MultiDimensional Scaling) of the different studied rims in Corsica and at Marseille, according to the protection level (WR, within reserve—red dots; outside reserve—black dots) and the circle of correlations of the different descriptors (categories). The rims with the name in pink are located near the mean sea level, those in green are a few tens of centimeters above the sea level and those in blue are far above sea level, 50 to 100 cm). Processing: Square root; Similarity Matrix: S17 Bray Curtis; 2D Stress: 0.09. (**B)** dbRDA (Distance-based redundancy analysis) of the different studied rims in Corsica and at Marseille, according to the LBRHI index (in yellow LBRHI index > 0.25, in orange: index between 0.25 and 0.5, and in green: LBRHI index > 0.5) and correlations of the different descriptors (categories). The rims with the name in pink are located near the mean sea level, those in green are a few tens of centimeters above the sea level and those in blue are far above sea level, 50 to 100 cm). Processing: Square root; Similarity Matrix: S17 Bray Curtis.

### Do marine protected areas matter?

Both at Marseille and in Corsica, the LBRHI Index of the rims was higher within the core of the Reserve of both AMPs (M5 through M8, 0.48; C1 through C6, 0.23) than at other sites (M1 through M4, 0.20; C7 through C11, 0.11) (Table 2). Obviously, parameters other than the level of protection account for the differences between sites and regions (Fig. 4A, Tables 4, 5 and 6). The Distance-based redundancy analysis (dbRDA) shows that 81.3% of total variation is explained by the first two axes, bdRDA1 and bdRDA2 (Fig. 4B).

**Table 4:** Permutation multivariate analysis of variance (PERMANOVA) on square root transformation of the different studied rims, according to the protection level, hydrodynamism and their height above mean sea level, using S17 Bray Curtis similarities. No significant value was found.

	*df*	SS	MS	Pseudo-F	P (perm)	Unique perms
Protection Level	1	1163.5	1163.5	3.5361	0.037	998
Hydrodynamism	1	224.04	224.04	0.68088	0.54	998
Height above mean sea level	2	563.42	281.71	0.85615	0.518	999
Protection Level x Hydrodynamism	1	19.855	19.855	0.060341	0.968	999
Protection Level x Height above mean sea level	2	685.52	342.76	1.0417	0.386	998
Residual	11	3619.5	329.04			
Total	18	6275.8

**Table 5:** Relationship between the Health Index (LBRHI) of the studied rims and their position above the mean sea level. See Table 1 for study sites localization.

	Height above mean sea level	Sites	Mean Health Index (LBRHI)
Calanques National Park (CNP) (Marseille)	Low	M1, M6, M7, M8	0.49 ± 0.08
	High	M5	0.30
	Very high	M2, M3, M4	0.15 ± 0.02
Scandola Nature Reserve (SNR) and surrounding area (Corsica)	Low	C2, C4, C5, C7, C8, C11	0.17 ± 0.04
	High	C1, C3, C9, C10	0.16 ± 0.04
	Very high	C6	0.32

**Table 6:** Student’s t test between each case of rim position above the mean sea level. When the differences are statistically different at p = 0.001 and p = 0.01, they are represented by ***. and **, respectively, and NS if the differences are not statistically different.

	Position above the mean sea level	|t| calculate	df	*p* value
Calanques National Park (CNP) (Marseille)	Low/High	4,75**	3	0,01
	Low/Very high	8,17***	5	0,001
	High/Very high	12,99**	2	0,01
Scandola Nature Reserve (SNR) and surrounding area (Corsica):	Low/High	0,39^NS^	3	
	Low/Very high	-9,19***	5	0,001
	High/Very high	-8,00**	3	0,01

### Does the height of the rim above sea level matter?

**Figure 5:**
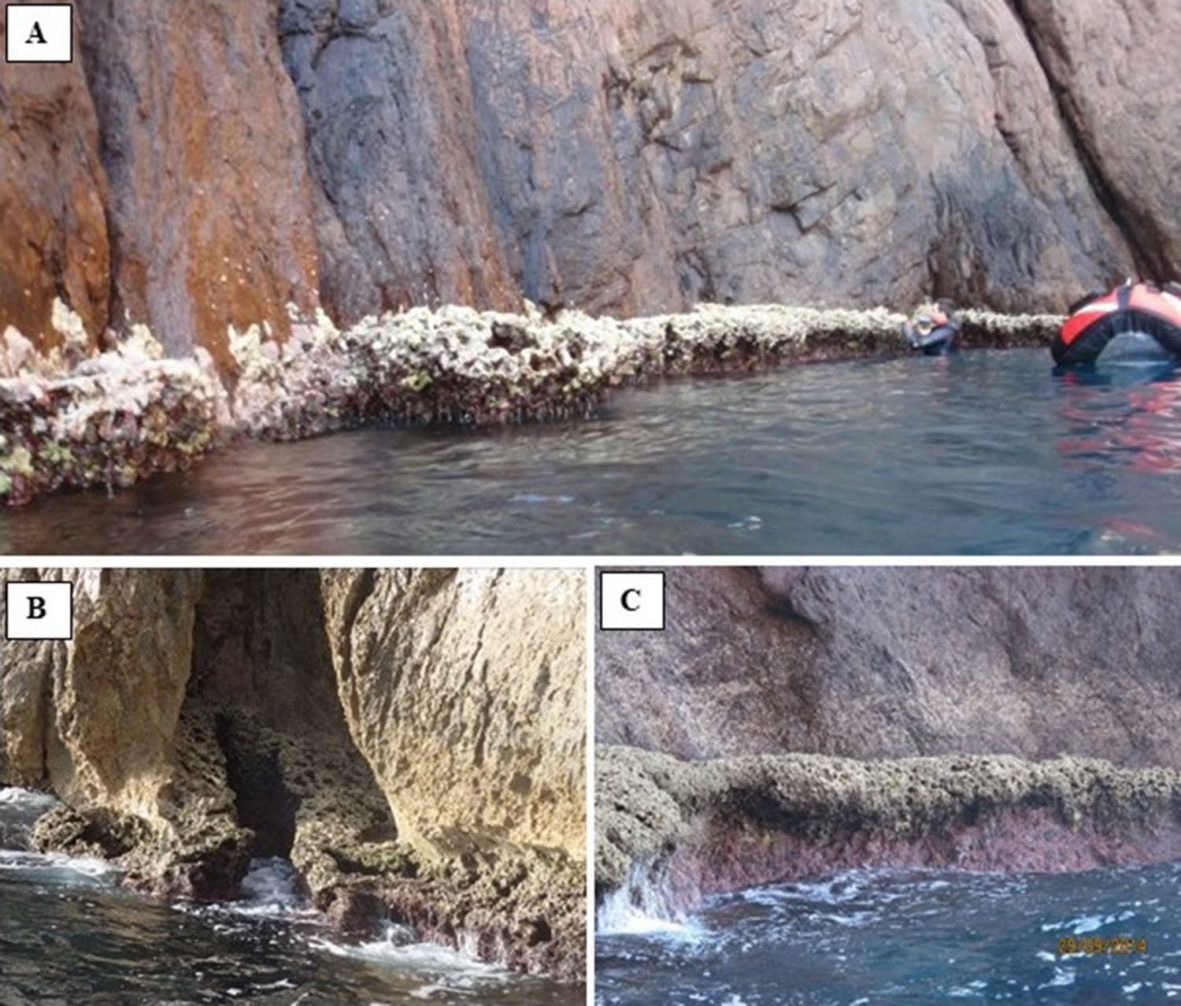
Height of the *Lithophyllum byssoides* rim above the mean sea level. (**A**) Low (Site C1, Palazzu cave). (**B)** high (Site C3, Punta Palazzu). (**C)** very high (site M2, Calanque de l’Oule). Photos © Aurélie Blanfuné and Thierry Thibaut.

Depending upon the relatively calm or exposed conditions, and the topography of the shore, the upper surface of the *L. byssoides* rim can be close to the mean sea level (20–30 cm), far above it (30–50 cm) or very far above it (up to 100 cm) (Fig. 5). While the surface of the lowest rims is more or less horizontal, parallel to the sea level, the surface of the two other categories of rims are sloping towards the sea almost vertically.

The vitality of *L. byssoides* rims could linked to its height above the sea level, considering the sensitivity of this midlitoral ecosystem to submersion. The rims located well above the mean sea level are likely less often immersed, but also exposed to sea surface pollutants and bioerosion, than those close to sea level. However, comparison of the LBRHI values of low, high, and very high rims, in the two study regions, does not show any significant differences, ruling out any major driving effect of the closeness to sea level (Tables 4, 5 and 6).

## Discussion

The LBRHI index is very heterogeneous between sites within a region, sometimes between relatively close sites (e.g. C1 and C6). These heterogeneous results are similar to those obtained north of the SNR, in September 2011: at Punta di Vigatoggiu, dead *L. byssoides* ranged between 70 and 80% of the cover and numerous holes were present (between 14 and 22%). Rims at Punta di U Ciuttone showed a cover of 64% of live *L. byssoides* on the western rim vs. 13% on the eastern rim^43^. Alarmingly, during the 2014–2015 field survey covered in this study, the ‘honeycomb-like appearance’, with densely interweaving and anastomosing cristae, a sign of good vitality^26^, was never observed.

By its exceptional dimensions (up to 2 m wide), the *L. byssoides* rim at Cala Litizia (Corsica) has been recognized as probably unique since the establishment of the SNR^44^. It has been extensively and regularly surveyed over time, allowing a comparison of its successive states and an assessment of its evolution^13,18,20,21,45,46^. From 1976–1978 to 1995, the contours of the rim (outer edge) seem to have remained more or less stable^44^. In contrast, its health status conspicuously declined over time. In 1976–1978, shortly after the establishment of the SNR, the rim was reported to be in very good condition^18^. Between 1981 and 1986, Bianconi et al.^13^ made no allusion to possible traces of erosion while pictures from that time reveal traces of degradation on the front of the *L. byssoides* rim. Between 1987 and 1992, Laborel et al*.*^20,46^ observed a clear and rapid decline: they estimated the average coverage of live *L. byssoides* at less than 50% and noted numerous marks of impact and erosion, deep breaks over 5 cm thick, traces of mooring ropes and the appearance of erosion pits in the range of 20 to 30 cm in diameter.

Marine protected areas (MPAs) have often been established in areas little impacted by humans, e.g. because of historical features, their insularity and/or their remoteness. This is the case of the Port-Cros National Park in eastern Provence^47^ and of the Scandola Nature Reserve (SNR) in western Corsica^38^. These features can induce a bias and create the illusion that a good state of health results from the protection, whereas it is simply due to the remoteness of the MPA and the historical absence of human impact. However, many MPAs have been established in areas where human impact is strong, or has been high in the near past. This is the case with the Calanques National Park (CNP), between Marseille and La Ciotat (western Provence). In addition to the presence in the immediate vicinity of Marseille, one of the oldest (sixth century BCE) and most populous cities and ports in the western Mediterranean, a sewage outfall discharged untreated wastewater from 1896 to 1987 into the core area of what is since 2012 the CNP. The physical and chemical treatment plant was commissioned in 1987 and the biological treatment plant only in 2007. At Marseille, it is unlikely that the effect of protection, for such a long-living and slow-growing formation, would be noticeable only 3 years after the establishment of the CNP. In Corsica, the general low level of the LBRHI index, after 40 years of effective protection (the SNR is anything but a ‘paper park’- 38), and its sharp decline over time at Cala Litizia (site C2), where historical data exist (Table 3), does not suggest a positive effect of protection.

The monitoring of the *L. byssoides* rim of Cala Litizia over 40 years, by the same research team with some recurrent researchers (CFB and MV), rules out any potential bias linked to methodological changes. In addition, the *L. byssoides* rims of the SNR have been fully mapped three times, in 1981–1986^13^, in 1993–1996^49^ and in 2007–2012^32,34–36^, along the 39 km of coastline of the reserve (at the scale considered: 1/2500), along stretches of 20 m and 50 m, respectively. Hence, in a scientific world lacking temporal baselines for biological entities^50^, this available extensive baseline represents a unique resource for tracking change in ecosystems in the human-altered Mediterranean Sea. In the face of the alarming decline of the *L. byssoides* rims occurring at large scale in the northwestern Mediterranean Sea, it is therefore legitimate to review the possible causes of such changes, especially since the degradation trend does not seem to be local, but concerns a vast biogeographical area.

The first source of degradation could be due to mechanical attacks which correspond to friction and shocks that scratch and destroy the thin surface layer of live *L. byssoides*. They can be caused by exceptional storms, boats, landings or trampling, but can also be due to floating macro-waste trapped in the creeks (e.g. driftwood, plastics) as well as rockfall. In Corsica, the *L. byssoides* rim of Cala Litizia (site C2) (Fig. 2) appears in recent tourist guides and has featured in the tour program of several tour operators (‘Corsica tour in inflatable boat’); marks of friction of boats and their mooring ropes, when these boats try to unload tourists on the *L. byssoides* rim, then of trampling, were visible^21,26,38^. In contrast, in narrow coves of the SNR, where access and disembarkation are difficult, only very few signs of mechanical deterioration were noticed, although some rims were not in good health (e.g. Gattaghja South)^21,51^.

A second source of degradation could be chemical pollutants, which can come from the air (rainfall), the land and the sea. Several authors have pointed out the regression of *L. byssoides* rims in polluted areas^52,53^. In the CNP, the main sources of marine pollution were, from the late nineteenth century to the late 20st century, the Huveaune River, which flows into the sea in the south of the bay of Marseille, and the Cortiou sewage outfall, which discharged untreated sewage from 1.5 million equivalent inhabitants from 1896 to 1987. Today, following the commissioning of the physical and chemical treatment plant in 1987 and the biological plant in 2007, the relatively purified water from the wastewater treatment plant (WWTP) still flows into the core of the CNP. Impacts of rainfall and boats, both leisure and commercial, are also to be considered as a source of chemical-induced degradation. The absence of extensive blooms of Ulvales (often nitrophilic green algae) however makes it possible to rule out the hypothesis of a severe eutrophication caused by continental inputs. *Lithophyllum byssoides* being a calcified organism, an alteration due to atmospheric pollution and the resulting ‘acid rain’ could be possible, but the good state of the rims located highest above sea level (see below) allows this hypothesis to be rejected. In Corsica, a sparsely populated island, devoid of polluting industries, the RNS is further away from the main towns and river mouths; pollution is therefore a priori low.

**Figure 6:**
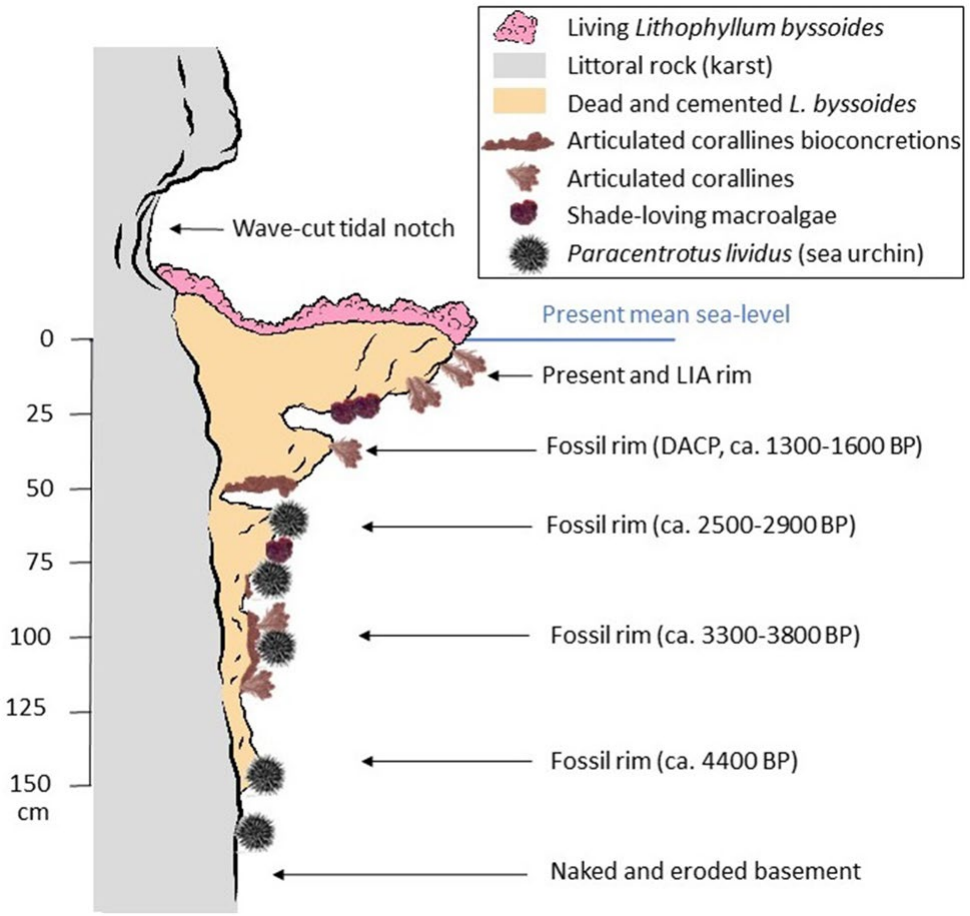
Profile of a cliff showing the structure of the present *Lithophyllum byssoides* rim (LIA—Little Ice Age—and post-LIA; DACP—Dark Ages Cold Period) and the remains of the previous submerged rims that are gradually disappearing under the influence of infralittoral bioerosion. From Laborel & Laborel-Deguen^51^, redrawn and simplified.

The fate of a *L. byssoides* rim depends on the balance between bioconstruction by *L. byssoides* and other crustose corallines, and bioerosion by endoliths (cyanobacteria and green algae), borers (bivalves, sponges) and grazers [e.g. the sea urchin *Paracentrotus lividus* (Lamarck, 1816)]^54^. If the balance is positive, the *L. byssoides* rim grows seawards and, if the sea level is gently rising, it grows upwards. Otherwise, the *L. byssoides* rim erodes. The activity of these bio-eroders is positively enhanced by wetting, as evidenced by the relatively rapid (centuries or millennia) disappearance of fossil rims, when they became submerged, due to the sea level rise^2,4,23,55^ (Fig. 6). The presence of boats sailing close to the coast, and the waves they generate, even in calm seas, contributes to dampening the *L. byssoides* rims and thus also facilitates the activity of bioeroders.

Emissions of CO_2_ and other greenhouse gases continue to increase over time^56,57^. While the rise in sea level was continuous during the Holocene^58,59^, since the beginning of the twentieth century we have witnessed global warming and a relatively rapid (less than a century) and significant (> 10 cm) increase in the speed of sea level rise (ca. 3 mm an^−1^ since the 1990s)^60–63^, and the rate is currently steadily growing^64–66^. As early as during the 1990s, Laborel et al.^14^ attributed the unexpected installation of individuals of *L. byssoides* 20 to 40 cm above the upper surface of some *L. byssoides* rims to the twentieth century rise in sea level (see also for more recent data,^23,51^). In the hypothesis of a further rise, according to Laborel et al. ^3,14^, no *L. byssoides* rim would have the capacity to survive a too rapid sea level rise. This upwards shift of the infralittoral zone accounts for the presence of infralittoral algae (COR and RH) on the edge and the upper surface of many *L. byssoides* rims, and for the fact that the lowest rims are often already submerged, with a sea level where *L. byssoides* cannot thrive (Fig. 5). Few elements are known about some aspects of the biology of *Lithophyllum byssoides* that are fundamental to predict its responses to environmental stressors, especially with regard to its genetic structure at population level^67^. It cannot be excluded that different populations living in different geographical areas might respond differently to climate-related changes, and some populations (for example in the eastern Mediterranean) might turn out to be more resistant than currently believed^67^.

The rise in sea level cannot be controlled by local management; only the global reduction of greenhouse gases and the implementation of the Paris agreement on climate change of 2015^68^ could slow down—not stop—the sea level rise.

It is important to stress that the rise in sea level, from its position 120–130 m below the current level, about 20 000 years ago (Last Glacial Maximum—LGM), has been continuous over the entire period, with accelerations to 4 m per century, and a slowing down for 6 000 years^59,63,69^. This 'natural' rise should continue, since, at the end of the interglacial periods that preceded the current interglacial, it was between + 1 and + 9 m above the current level^48,63,70,71^. During periods of rapid rise, the *L. byssoides* rims probably disappeared, although the species itself has of course survived. The predicted disappearance of the current rims, due to the natural rise in sea level and its acceleration under the effect of human activities and global warming^22,23^, is irreversible on a human scale, not on a geological timescale.

## Conclusions

*Lithophyllum byssoides* is a very common midlittoral macroalga in the western Mediterranean Sea. Locally, under exposed and dim light conditions, *L. byssoides* builds unique formations (*L. byssoides* rims) whose height above mean sea level and width vary considerably.

Since the 1980s, the condition of these bioconstructions has deteriorated: poor coverage of living individuals of *L. byssoides*, invasion by infralittoral algae, bioerosion leading to the formation of holes that enlarge over time. The decline in its health has been observed at many sites far apart from each other, both in areas heavily impacted by humans and in areas with little impact, in MPAs as in unprotected areas.

The deterioration is too general to be attributed solely to local stressors. The most worrying stressor is the rise in sea level. Although sea level rise is a slow, long-term process, a sort of tipping point seems to have been reached. In contrast with other stressors, the rise in sea level has the particularity of not being able to be solved by local management.

The media and scientists often rightly draw the attention of the public to the flooding of Pacific atolls and the States that are affected as a result. It is also worth drawing attention to the fate of the Mediterranean *L. byssoides* algal rims, an ecosystem endemic to the Mediterranean Sea, and to its possible forecast collapse; if so, it would be the first case worldwide of marine ecosystem collapse resulting, indirectly, from global warming and man-induced global change.

## Data Availability

All data generated or analysed during this study are included in this published article.
